# A Study of Transplacental Transfer of SARS-CoV-2 Antibodies in COVID-19 Vaccinated Women in Mumbai, India

**DOI:** 10.7759/cureus.87643

**Published:** 2025-07-10

**Authors:** Disha Bhanushali, Rahul Verma, Shreyans Rai, Shashikala Shivaprakash, Dipti Dhanwate, Maninder S Setia

**Affiliations:** 1 Pediatric Medicine, Sir H. N. Reliance Foundation Hospital and Research Centre, Mumbai, IND; 2 Biostatistics, Sir H. N. Reliance Foundation Hospital and Research Centre, Mumbai, IND; 3 Microbiology, Sir H. N. Reliance Foundation Hospital and Research Centre, Mumbai, IND; 4 Epidemiology, Sir H. N. Reliance Foundation Hospital and Research Centre, Mumbai, IND

**Keywords:** anti-nucleocapsid antibodies, anti-spike antibodies, covishieldtm vaccine, neutralizing antibodies, transplacental transfer

## Abstract

Background

Antibodies generated in response to vaccination in mothers are efficiently transferred across the placenta. The transfer of these antibodies may depend on the time of vaccination during pregnancy, the type of vaccine, and the number of doses, including booster doses. We designed the present study to study the correlation between anti-spike (anti-S), anti-nucleocapsid (anti-N), and neutralizing antibodies in vaccinated mothers and neonates in Mumbai, India. We also wanted to evaluate the factors affecting the level of antibodies in mothers and neonates.

Methods

The present study is a cross-sectional analysis of 125 mother-neonate dyads in Mumbai, India. We recruited a consecutive consenting sample of mothers who were in the period of 37 to 42 weeks. We collected blood from the mothers just prior to delivery, and cord blood was used for testing in newborns. In addition, we also collected information on demographics, pregnancy, COVID-19 history, and vaccination details. We performed tests for three types of antibodies in these two types of blood samples for each dyad: anti-S, anti-N, and neutralizing antibodies.

Results

The mean (SD) age of the women was 28.8 (3.5) years. Most of them were at 37 weeks of gestation (56%), and 44% were at 38 or 39 weeks of gestation. All the women had taken two doses of the Covishield™ vaccine, and the mean (SD) duration between the second dose and date of delivery was 14.1 (1.4) months. The median (IQR) value of anti-S antibodies was 3012 (1555, 5000) U/ml in the mother, and it was 3749 (1636, 5000) U/ml in the cord blood. There was 100% reactivity to the anti-N antibodies in the mother and the cord blood (based on the cut-off index). There was 85.6% (n=107) positivity for neutralizing antibodies in the mother and 82.4% (n=103) positivity in the cord blood. The transfer ratio of > 1 was seen in 37.6% for anti-S antibodies and 51.2% for neutralizing antibodies; it was significantly higher for neutralizing antibodies compared with anti-S antibodies (p=0.03). In linear regression models, an increase of one unit of anti-S antibody in the mother was associated with an increase of 0.95 (95% confidence interval [CI]: 0.89, 1.00; p<0.001) U/ml in the baby. The positivity for these antibodies was significantly lower in the cord blood with an increase in the duration of the last dose of the vaccine in the mothers (odds ratio: 0.57, 95% CI: 0.34, 0.95; p=0.03).

Conclusions

We found that there was a significantly high positive correlation between the levels of COVID-19 antibodies in vaccinated mothers and the cord blood of neonates. Time since vaccination reduced the levels of antibodies in mothers; however, the drop per month was significant only for anti-S antibodies. The time of vaccination in the mothers was significantly associated with positivity for neutralizing antibodies in the cord blood; the positivity reduced with an increase in duration between the second vaccine and birth. There was no significant association between the age of the mother or gestation age and antibody levels in the cord blood.

## Introduction

Towards the end of 2019, the World Health Organization discussed media reports on cases of viral pneumonia in Wuhan, China, and a pandemic of coronavirus disease of 2019 (COVID-19) was declared in March 2020 [1]. The virus was named severe acute respiratory syndrome coronavirus 2 (SARS-CoV-2) [2]. Initially, management of COVID-19 infections included multiple medications and therapies, such as antibiotics, antivirals, oxygen therapy, steroids, biologics, or interferons [3-11]. An important addition to the management of this pandemic was the introduction of vaccines. There were multiple categories of vaccines: mRNA-based vaccines, viral vector-based vaccines, plasmid DNA-based vaccines, recombinant protein-based vaccines, and inactivated vaccines [12]. Many vaccination programs initially prioritized at-risk and vulnerable populations, such as health care workers, the elderly, and individuals with comorbidities [13]. Eventually, these programs covered the whole population.

Even though it has been reported that these vaccination programs reduced the spread of the virus and the clinical severity of the infection, there was hesitancy in acceptance of the vaccine [14,15]. This hesitancy was seen particularly in extremes of age (pediatric and geriatric populations), pregnant women, or those with autoimmune conditions or with an immunocompromised state; this hesitancy may be due to perceived side effects of the vaccine [16,17]. Some reasons for vaccine hesitancy in pregnant women were long-term effects on the baby, effects on breastfeeding, and concerns about side effects [18,19]. Nonetheless, vaccines were recommended in pregnant women and in those who were breastfeeding; the uptake in pregnant women ranged from 7% to 69%, and a meta-analysis reported a median uptake of 27.5% [20-24]. An important aspect of vaccination in pregnancy is the transplacental transfer of antibodies from the vaccinated mothers to neonates. It has been reported that the IgG antibodies generated in response to vaccination in mothers are efficiently transferred across the placenta [25-27]. These antibodies are known to persist in the newborn and may provide protection to them [28]. The transfer may depend on the time of vaccination during pregnancy, the type of vaccine, and the number of doses, including booster doses [25,27,29,30]. Furthermore, it has also been shown that even though there may be transfer of antibodies, the mRNA vaccine products themselves do not cross the placental barrier [25]. In India, the common vaccines used were Covishield™ (the Oxford/AstraZeneca vaccine manufactured by the Serum Institute of India) and Covaxin® (Bharat Biotech, India). The former is a ChAdOx1 nCoV-19 (Covishield™) recombinant vaccine, and the latter is an inactivated whole virion BBV-152 (Covaxin®) vaccine [31,32]. Few studies have reported the transplacental transfer of antibodies in pregnant women vaccinated with these Indian vaccines (the latter being completely indigenous).

Thus, we designed the present study to study the correlation between anti-spike (anti-S), anti-nucleocapsid (anti-N), and neutralizing antibodies in vaccinated mothers and cord blood of neonates born to these mothers. In addition, we will assess the transfer ratios of these antibodies and factors associated with antibody levels in mothers and the cord blood.

## Materials and methods

The present study is a cross-sectional analysis of 125 mother-neonate dyads in Mumbai, India.

Study site and population

The present study was conducted in a tertiary care center in South Mumbai, India. The data were collected over a period of three months (mid-October 2022 to mid-January 2023) after obtaining approval from the Ethics Committee. All mothers who had registered for delivery in this hospital were eligible for participation in the study. We recruited a consecutive consenting sample of mothers who were in the period of 37 weeks to 42 weeks of gestation. All mothers, irrespective of the mode of delivery, were included in the study. However, we excluded mothers who had reported positivity for COVID-19 two weeks before birth or had tested positive for COVID-19 at the time of delivery. We did a nasopharyngeal reverse transcription polymerase chain reaction test for COVID-19. The mothers did not report any COVID infection during pregnancy, and we did not have any documented reports of infection.

Study procedures

All participating mothers were explained the study procedures in detail. We collected blood from the mothers just prior to delivery, and cord blood was used for testing in newborns. In addition, we also collected information on demographics, pregnancy, COVID-19 history, and vaccination details. We performed tests for three types of antibodies in these two types of blood samples for each dyad. We used the Elecsys® anti-SARS-CoV-2 S (Hoffman-La Roche, Roche Diagnostics) test for quantitative assessment of antibodies to the spike protein (referred to as anti-S antibodies in the manuscript) [33]. This is an immunoassay to measure the total antibodies to SARS-CoV-2 S protein in the serum and plasma. This test provides the values of anti-S antibodies in U/mL. We used the Elecsys® anti-SARS-CoV-2 (Hoffman-La Roche, Roche Diagnostics) for measurement of total antibodies (including IgG) to the nucleocapsid (N) protein (referred to as the anti-N antibodies in the manuscript). The sandwich assay uses the antigen that represents the nucleocapsid protein. This test gives a cutoff index (COI) value; a COI of > 1.0 is reactive [34]. Finally, we used the GENLISA™ SARS-CoV-2 (COVID-19) surrogate virus neutralization test (sVNT)/neutralizing antibody ELISA test (KRISHGEN BioSystems, USA) for the detection of neutralizing antibodies. This test is used for qualitative and quantitative estimation of neutralizing antibodies in the serum and plasma. A COI of >0.5 was considered positive for neutralizing antibodies [35].

Statistical methods

Data were entered in MS Excel (Microsoft, USA) and analyzed using Stata Version 17 (© StataCorp, College Station, Texas, USA). We estimated the means and standard deviation (SD) for normal continuous data and median and interquartile range (IQR) for non-normal continuous data. We estimated the correlation between two linear variables using Pearson’s correlation coefficient. The transfer ratio was calculated as a ratio of the cord blood values to the maternal values. [36] We used regression models for multivariate analysis. For linear variables (level of antibodies), we used linear regression models, and for categorical variables (reactive/non-reactive), we used logistic regression models. For linear regression and logistic regression models in the mothers, the covariates included in the model were age of the mother, gestational age, and time from the last vaccine dose (per month increase). For the linear regression models in the cord blood, the covariates in the model were antibody levels in the mother (per unit increase), age of the mother, gestational age, and time from last vaccine dose (per month increase). For the logistic regression models in the cord blood, the covariates included in the model were antibody status in the mother (positive/negative), age of the mother, gestational age, and time from the last vaccine dose. A p-value of <0.05 was considered statistically significant.

The study was approved by the Institutional Ethics Committee of Sir H N Reliance Foundation Hospital and Research Centre (Reference No. IEC Protocol No. IEC/2022/DNB/PED/22 Date: 07 October 2022). The study was conducted according to the principles of the Declaration of Helsinki, and the mothers provided written informed consent for participation.

## Results

The mean (SD) age of the women was 28.8 (3.5) years. Most of them were at 37 weeks of gestation (56% [n=70]), and 44% (n=55) were at 38 or 39 weeks of gestation. All the women had taken two doses of the Covishield™ (Oxford/AstraZeneca, manufactured by Serum Institute of India) vaccine. The mean (SD) duration between the second dose and date of delivery was 14.1 (1.4) months.

**Figure 1:**
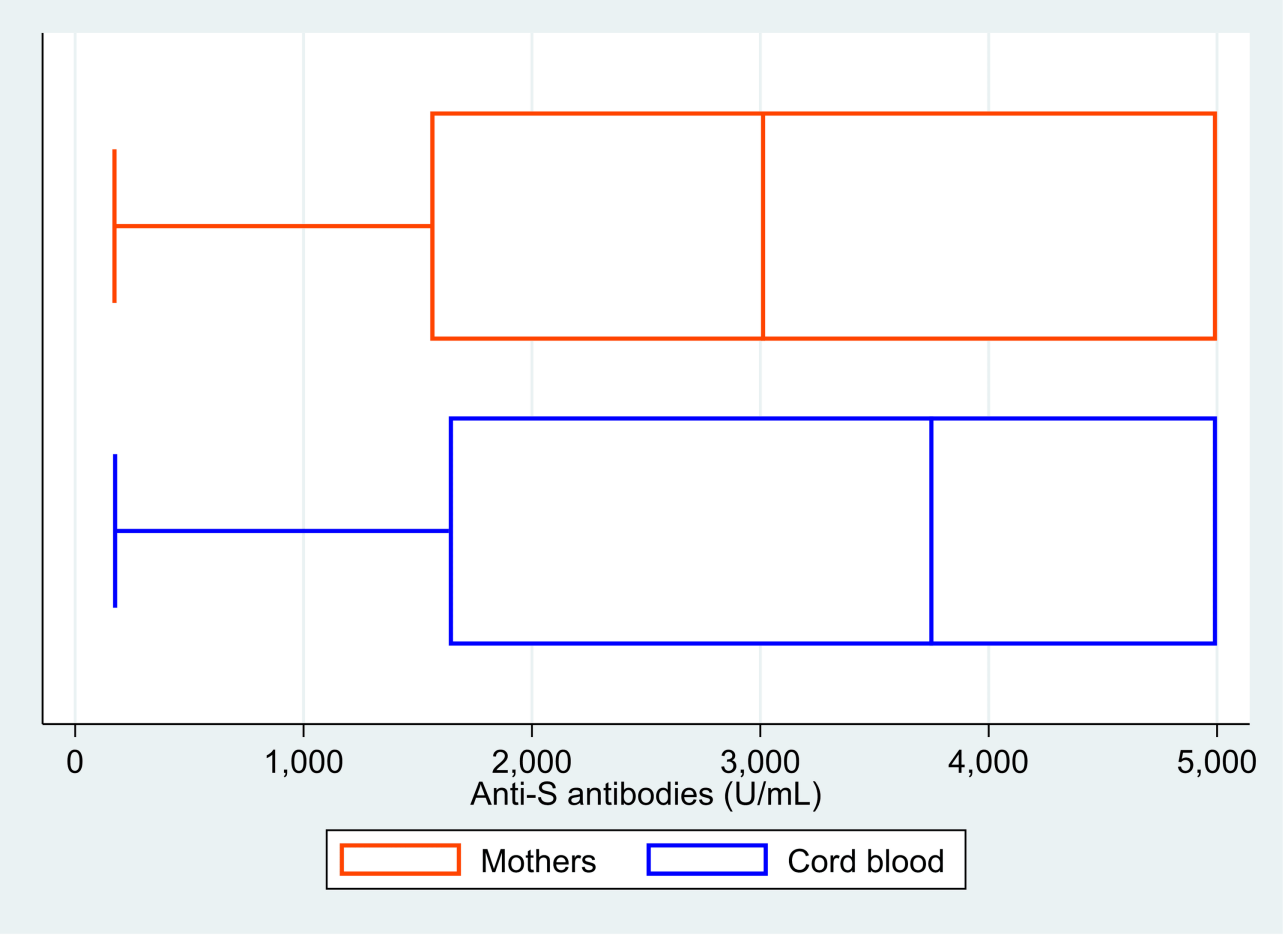
Box plot showing the levels of anti-spike (anti-S) antibodies in 125 mother and neonate dyads, Mumbai, India. The image is created by the author.

**Figure 2:**
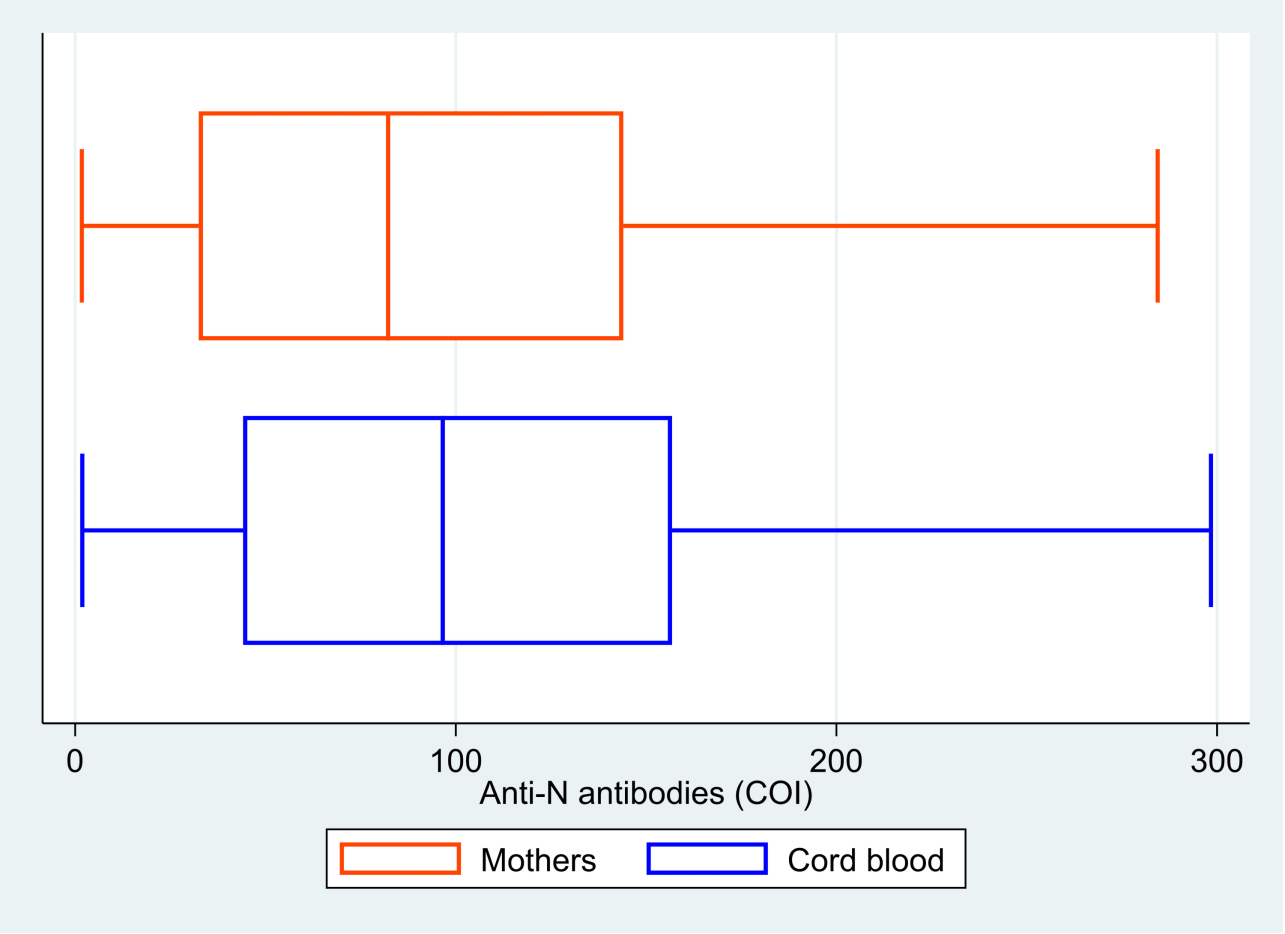
Box plot showing the cut off index levels of anti-nucleocapsid (anti-N) antibodies in 125 mother and neonate dyads, Mumbai, India. The image is created by the author.

**Figure 3:**
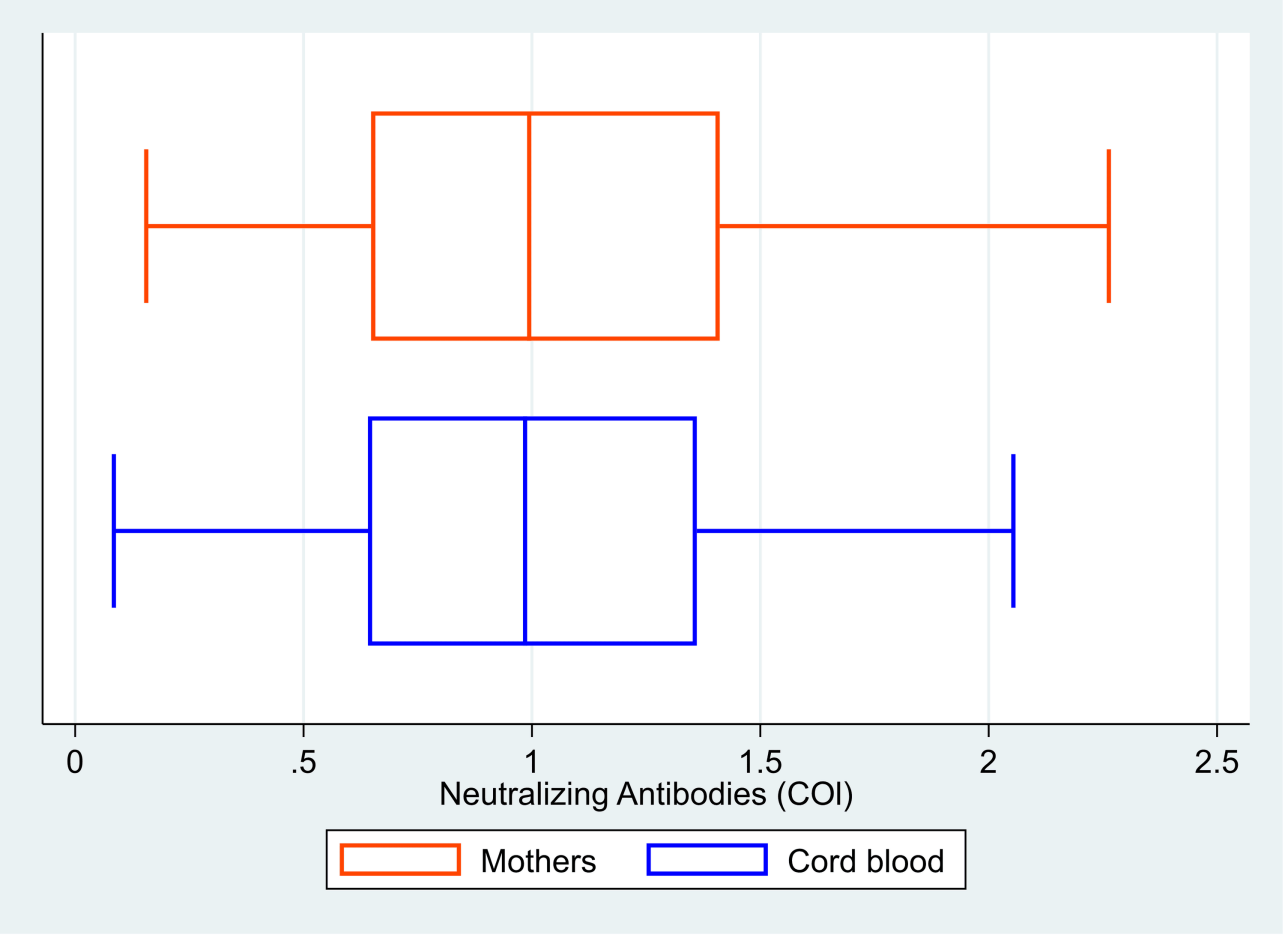
Box plot showing the cutoff index levels of neutralizing antibodies in 125 mother-and-neonate dyads, Mumbai, India. The image is created by the author.

**Figure 4:**
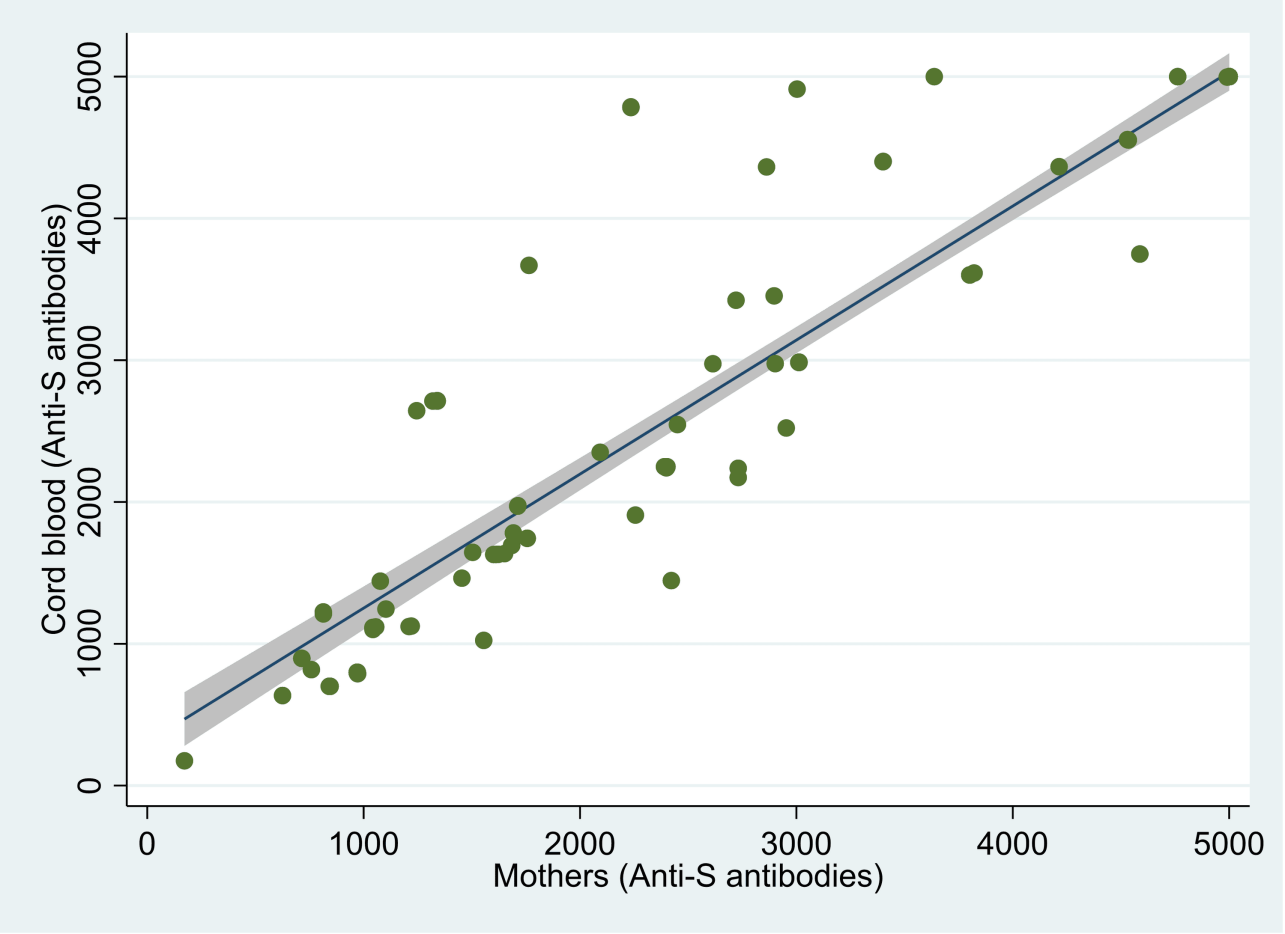
Scatter plot of levels of anti-spike (anti-S) antibodies in 125 mother and neonate dyads, Mumbai, India. The image is created by the author.

**Figure 5:**
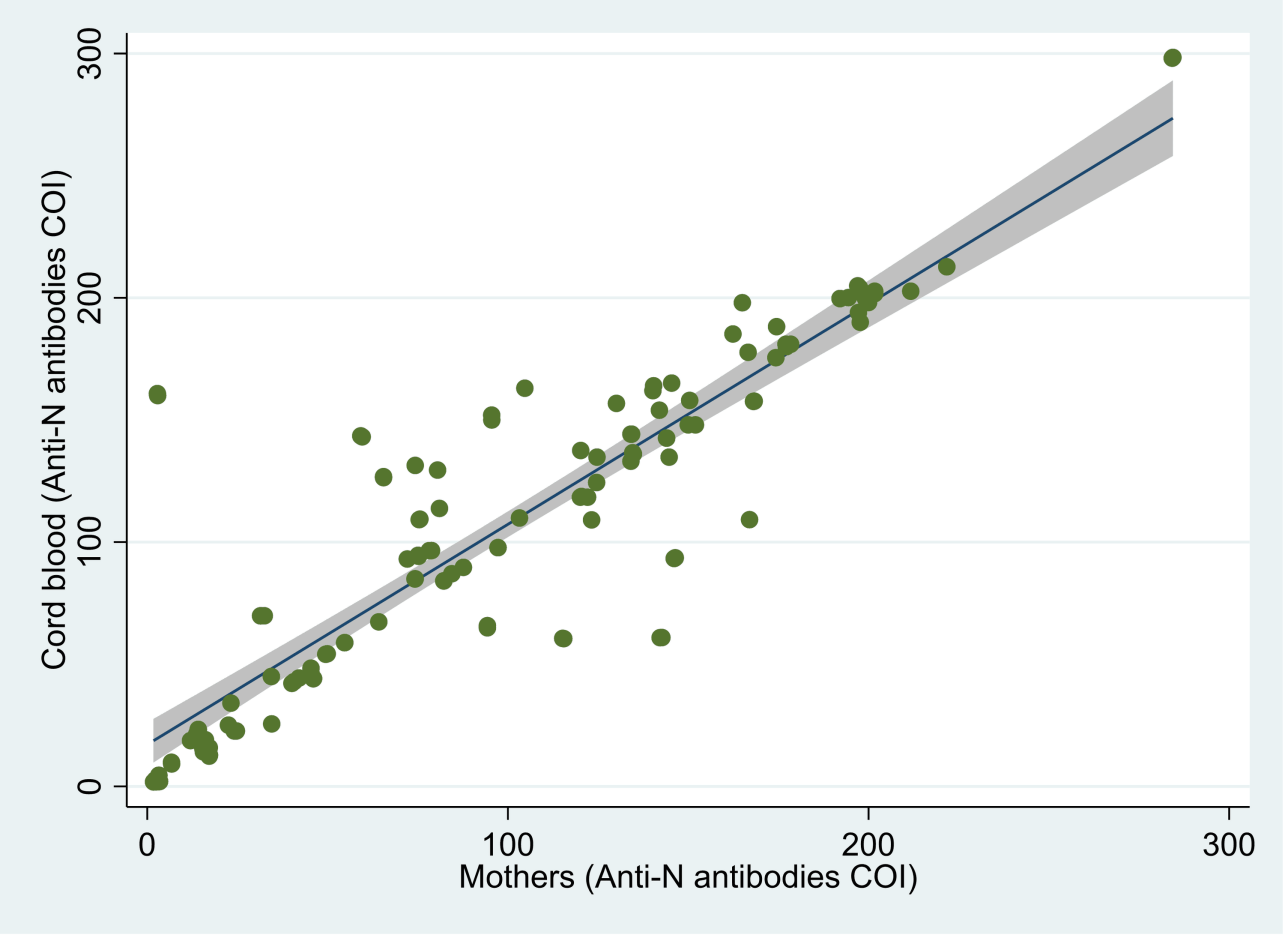
Scatter plot of cutoff index for anti-nucleocapsid (anti-N) antibodies in 125 mother and neonate dyads, Mumbai, India. The image is created by the author.

**Figure 6:**
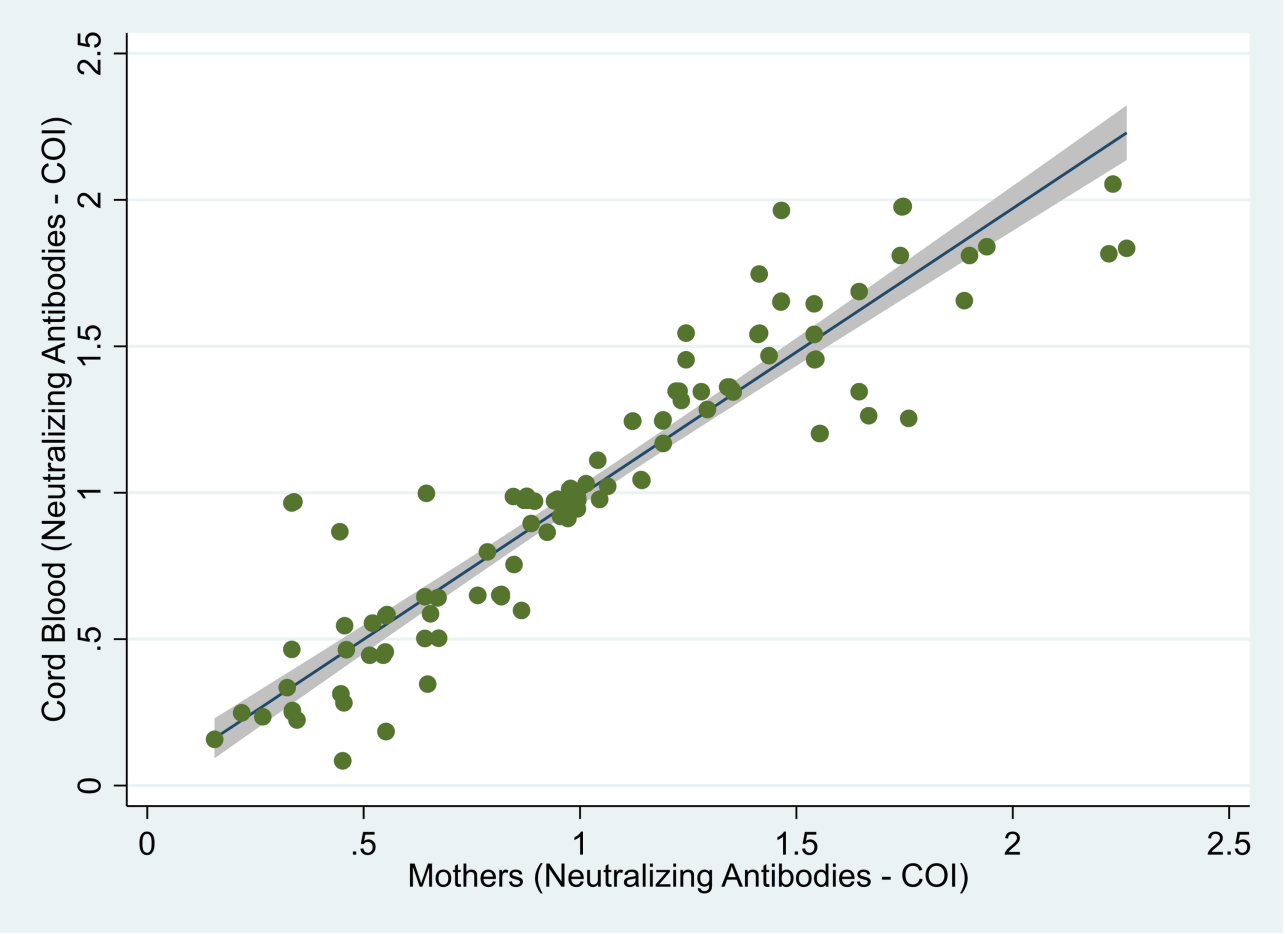
Scatter plot of levels of neutralizing antibodies in 125 mother and neonate dyads, Mumbai, India. The image is created by the author.

The median (IQR) value of anti-S antibodies was 3012 (1555, 5000) U/ml in the mother, and it was 3749 (1636, 5000) U/ml in the cord blood (Figure 1). The median (IQR) COI value for anti-N antibodies was 82.2 (32.5, 144.0) in mothers and 96.6 (44.1, 156.8) in cord blood (Figure 2). There was 100% reactivity to the anti-N antibodies in the mother and the cord blood (based on the cut-off index). The median (IQR) COI values for the neutralizing antibodies in the mother and the cord blood were 0.99 (0.65, 1.41) and 0.99 (0.64, 1.36), respectively (Figure 3). All the median values are presented in Table 1. There was 85.6% (n=107) positivity for neutralizing antibodies in the mother and 82.4% (n=103) positivity in the cord blood. There was a high correlation between the values of anti-S antibodies (r=0.95, p<0.001) in the mother and the cord blood. Similarly, there was also a significant correlation between COI for anti-N antibodies (r= 0.90; p<0.001) and neutralizing antibodies (r= 0.92; p<0.001) in the mother and the cord blood. Details of these values are provided in Table 2. The scatter plots for all three of these have been shown in Figures 4-6. The transfer ratio of > 1 was seen in 37.6% (n=47) for anti-S antibodies. The transfer ratio of >1 (according to the COI values) was seen in 51.2% (n=64) for neutralizing antibodies and 65.6% (n=82) for anti-N antibodies. In our data, efficient transfer was significantly higher for neutralizing antibodies compared with anti-S antibodies (p=0.03), and for anti-N antibodies compared with anti-S antibodies (p<0.001). Finally, efficient transfer was higher for anti-N antibodies compared with neutralizing antibodies (p=0.02).

**Table 1:** The median values along with interquartile range (IQR) of antibodies in 125 mother-neonate dyads, Mumbai, India. *p<0.001. The p-value was calculated using the Wilcoxon-Mann-Whitney test. A p-value of <0.05 was considered statistically significant.

Antibodies	Median (IQR) [Mother]	Median (IQR) [cord blood]	p-value
Anti-S antibody (u/ml)	3012 (1555, 5000)	3749 (1636, 5000)	0.046
Anti-N antibody (COI)	82.2 (32.5, 144.0)	96.6 (44.1, 156.8)	<0.001*
Neutralizing Antibodies (COI)	0.99 (0.65, 1.41)	0.99 (0.64, 1.36)	0.88

**Table 2:** Pearson’s correlation coefficient (r) values between the antibodies in 125 mother-neonate dyads, Mumbai, India. **p<0.01. The r value is Pearson’s correlation coefficient. A p-value of <0.05 was considered statistically significant.

Antibodies	r value	p-value
Anti-S antibody (U/ml)	0.95	<0.001**
Anti-N antibody (COI)	0.90	<0.001**
Neutralizing Antibodies (COI)	0.92	<0.001**

In the linear regression models for anti-S antibodies in the mothers, we found that mean antibody levels were lower in mothers whose gestational age was >37 weeks compared with a gestational age of 37 weeks; however, this was not statistically significant (-204.10, 95% confidence intervals [CI]: -808.60, 400.40; p=0.51). However, there was a significant decrease in the levels of the anti-S antibodies with an increase in the duration between the test and the last dose of the vaccine (-254.22, 95% CI: -475.17, -33.27; p=0.024). In the linear regression models for anti-S antibodies in cord blood, after adjusting for age of the mother, gestational age, and time from the second dose of vaccine, we found that an increase of one unit of anti-S antibody in the mother was associated with an increase of 0.95 (95% CI: 0.89, 1.00; p<0.001) U/ml in the cord blood. The levels of antibodies in the cord blood were lower in mothers aged >=30 years compared with those who were 20-29 years old; however, this was not statistically significant (-77.86, 95% CI; -279.76, 124.05; p=0.45). Even though there was a reduction in the levels of anti-S antibodies per month from the last vaccine in the cord blood, this was not statistically significant (-8.91, 95% CI: -81.11, 63.28; p=0.81). Detailed regression models have been presented in Table 3.

**Table 3:** Multivariate linear regression models for anti-spike (anti-S) in 125 mother-neonate dyads, Mumbai, India. *p<0.05, **p<0.01, CI: confidence intervals. The values indicate the estimate along with their 95% confidence intervals from the linear regression models. The first model is for the levels in the mother, and the second model is for the levels in the cord blood.

Parameters	Anti-S antibodies (mothers)		Anti-S antibodies (cord blood)	
	Estimate (95% CI)	p value	Estimate (95% CI)	p value
Antibody values in the mother				
Per unit increase	--		0.95 (0.89, 1.00)	<0.001**
Age of mother				
20-29	Reference	--	Reference	--
>=30 years	324.91 (-303.44, 953.25)	0.31	-77.86 (-279.76, 124.05)	0.45
Gestational Age				
37 weeks	Reference	--	Reference	--
> 37 weeks	-204.10 (-808.60, 400.40)	0.51	-25.11 (-218.88, 168.65)	0.80
Time from last vaccine dose				
Per month increase	-254.22 (-475.17, -33.27)	0.02*	-8.91 (-81.11, 63.28)	0.81

In the logistic regression models for neutralizing antibodies in the mothers, we found that even though positivity for these antibodies was lower in mothers aged >=30 years compared with those who were 20-29 years old, this was not statistically significant (odds ratio [OR] 0.51, 95% CI: 0.18, 1.40; p=0.19). There was no significant association between gestational age or time from the last dose of vaccine and positivity for neutralizing antibodies in the mothers. In the logistic regression models for cord blood, we found that positivity for neutralizing antibodies was significantly associated with positivity in the mothers (OR: 63.99, 95% CI: 13.41, >100; p<0.001). The positivity for these antibodies was significantly lower in the cord blood with an increase in the duration of the last dose of the vaccine in the mothers (OR: 0.57, 95% CI: 0.34, 0.95; p=0.03). Even though positivity was lower in babies born to mothers >=30 years, this association was not statistically significant (OR: 0.30, 95% CI: 0.08, 1.11; p=0.07). There was no significant association between gestational age and positivity for neutralizing antibodies in the cord blood. We have presented the logistic regression models in Table 4. We did not build any logistic regression models for anti-N antibodies since there was 100% positivity in the mothers and cord blood.

**Table 4:** multivariate logistic regression models for positivity for neutralizing antibodies in 125 mother-neonate dyads, Mumbai, India. * p<0.05, *p<0.001, ¶ p=0.07, CI: confidence intervals. The values are odds ratios along with their 95% confidence intervals. The first model is for antibody positivity in the mothers, and the second model is for antibody positivity in the cord blood.

Parameters	Neutralizing Antibody Positivity (Mothers)		Neutralizing Antibody Positivity (Cord blood)	
	Odds Ratio (95% CI)	p value	Estimate (95% CI)	p value
Mother Status				
Negative	--	--	Reference	--
Positive	--	--	63.99 (13.41, >100)	<0.001**
Age of mother				
20-29	Reference	--	Reference	--
>=30 years	0.51 (0.18, 1.40)	0.19	0.30 (0.08, 1.11)	0.07 ^¶^
Gestational Age				
37 weeks	Reference	--	Reference	--
> 37 weeks	0.96 (0.35, 2.66)	0.94	0.86 (0.24, 3.07)	0.82
Time from last vaccine dose				
Per month increase	1.00 (0.69, 1.45)	0.99	0.57 (0.34, 0.95)	0.03*

## Discussion

In our study, we found that there was a high correlation between the levels of anti-S antibodies for COVID-19 among vaccinated mothers and cord blood. Anti-N antibodies were positive for all mothers and neonates. There was a significant association between positivity for neutralizing antibodies in the mother and the cord blood. There was a significant reduction in the anti-S antibodies in the mothers with an increase in duration from the second vaccine. Similarly, positivity was lower in the cord blood with an increase in the duration of the second vaccine. Even though there was a reduction in the levels of anti-S and neutralizing antibodies in babies with a duration between the second vaccination and birth, this per-month reduction was not statistically significant. Babies born to older mothers (>=30 years) had lower levels of neutralizing antibodies. The transfer efficacy was highest for anti-N antibodies, followed by neutralizing antibodies and anti-S antibodies.

As seen in our study, there is a high correlation between the levels of COVID-19 antibodies in the mother and the cord blood. Proto and colleagues studied the presence of COVID-19 antibodies in mothers and neonates. They also found that there was a significant positive correlation between the anti-S antibodies in the mother and the child. This was seen in mothers who had received a single dose as well as two doses of the vaccine. In general, the titers were higher in both the mother and the child who had received both doses of vaccine compared with a single dose of the vaccine [37]. In this study, all the included women were vaccinated with mRNA vaccines (BNT162b2 [Comirnaty] and Moderna vaccines) [37]. Another study by Farhani and coworkers reported transplacental transfer of IgG antibodies in seropositive women [38]. They also found a high correlation between the antibody levels of seropositive mothers and neonates (correlation value of 0.96). Furthermore, in their study population, ‘efficient or higher transfer’ of antibodies was seen in nearly 55% of cases. Mothers with lower BMI and neonates with a higher weight had a higher transfer ratio of antibodies. Ateyo et al. also studied the transplacental transfer of antibodies in three different types of vaccine platforms (mRNA-1273, Ad26.COV2S, and BNT162b2) [29]. They found that spike-specific antibodies and Fc receptor binding were significantly higher in those who had received mRNA-1273 and BNT162b2 vaccine types compared with the Ad26.COV2.S type. In addition, they also found that higher anti-spike antibodies were found in cord blood if the vaccination in the mother was in the first and second trimesters, but not in the third trimester [29]. Our vaccine type was the ChAdOx1 nCoV-19 recombinant-type vaccine. We also found a high correlation between maternal and cord-level antibodies. A transfer ratio of > 1 is generally considered to be an efficient transfer [39]. In our study population, efficient transfer of antibodies was significantly higher for neutralizing antibodies and anti-N antibodies compared with anti-S antibodies.

We also assessed the relation of these antibodies and maternal and vaccination characteristics. We found that there was a significant reduction in the levels of anti-S antibodies in the mothers with an increase in the duration of the second dose of vaccine and birth; however, this was not significant for neutralizing antibodies. The positivity for neutralizing antibodies in the cord blood reduced with an increase in the duration between the second dose of vaccine in the mothers and birth. Other authors have also studied the factors associated with the transfer of antibodies to neonates. Shook and colleagues compared the transfer of anti-spike antibodies in women who were vaccinated versus those who had natural infection. They found significantly higher titers of antibodies in children born to vaccinated mothers compared with infected mothers; these antibodies persisted longer in vaccinated mothers as well [40]. Another study found that levels of antibodies were significantly lower in mothers and their babies who were vaccinated in the first trimester of pregnancy [37]. Furthermore, in this same study, they found that the levels of antibodies decreased over time from birth [37]. Wang and colleagues studied the transfer of antibodies in women infected with COVID-19 infection [41]. They found that the transfer of IgG antibodies was higher in infants if their mothers were infected for less than two weeks compared with those who were infected more than two weeks ago. Furthermore, they also reported that the IgM level in all infants was below the threshold of detection irrespective of the trimester of infection or time since infection [41]. Lee and co-workers studied the levels of anti-S and anti-N antibodies in individuals who were infected after vaccination; they reported that the levels of these antibodies were higher in the infected group compared with the non-infected group [42]. Flannery and colleagues compared the antibody levels in women who were vaccinated with those who were infected with SARS-CoV-2. They reported that though maternal and cord antibody levels were higher in those vaccinated compared with those infected, the transfer ratio of antibodies was lower in vaccinated individuals, and time from the event (either infection or vaccination) was associated with the efficiency of transfer of these antibodies [43]. Kachikis and coworkers reported that women who had received three doses of vaccine had significantly higher levels of anti-S antibodies compared with those who had received two doses only [44]. They did not find any association between the time of delivery (preterm or term) and the level of antibodies. Liu and colleagues have also studied the role of multiple factors in the transfer of antibodies to the neonates [45]. They found that transfer was higher in vaccinated women compared with infected women. However, they concluded that these antibodies provided limited protection against the infection in these infants.

Though previous studies have compared the number of doses, all the women in our study had received two doses of the vaccine (the recommended dose at that time). This provided uniformity of doses in our study. An important limitation of the study was that this was a cross-sectional study, and we measured the antibody levels only at one time (at birth in the cord blood). Due to the cross-sectional nature, we cannot comment on causality. Other authors have assessed the levels of antibodies longitudinally [40,41]. They found that levels of antibodies dropped to one-tenth at two months compared with birth levels [41], and nearly 43% of infants had no detectable antibody levels at six months after birth [40]. All the mothers had taken only one type of vaccine. Hence, we could not compare the transmission and efficiency of different types of vaccines that were approved in India (such as Covishield™ and Covaxin®); this was another limitation of the study. However, we did do multivariate analysis to account for time since vaccination, age of the mother, and gestational period. Furthermore, we also assessed all three types of antibodies (anti-S, anti-N, and neutralizing antibodies) in our study. In addition, though we tested the mothers for COVID-19 infection prior to inclusion, and we did not have any history of prior infection in them, we cannot rule out prior infection (not recorded or not detected), particularly within the perspective of 100% positivity for anti-N antibodies. In addition, with just one group, there may be limited generalizability of results. Thus, the results have to be interpreted within the constraints of these limitations.

## Conclusions

We found that there was a significantly high positive correlation between the levels of COVID-19 antibodies in vaccinated mothers and the cord blood of neonates. Time since vaccination reduced the levels of antibodies in mothers; however, the drop per month was significant only for anti-S antibodies. The positivity for neutralizing antibodies in the cord blood reduced with an increase in the duration between the second dose of vaccine in the mothers and birth. There was no association between gestational age and levels of anti-S antibodies or gestational age and positivity for neutralizing antibodies. The transfer ratio of >1 was significantly higher for anti-N antibodies and neutralizing antibodies compared with anti-S antibodies. Thus, babies born to mothers vaccinated for COVID-19 with the Covishield Vaccine™ had the presence of all three antibodies (anti-S, anti-N, and neutralizing antibodies).
